# Testing the Reliability of a Procedure Using Shear-Wave Elastography for Measuring Longus Colli Muscle Stiffness

**DOI:** 10.3390/s26010065

**Published:** 2025-12-22

**Authors:** Juan Izquierdo-García, Juan Antonio Valera-Calero, Marcos José Navarro-Santana, Ibai López-de-Uralde-Villanueva, Gabriel Rabanal-Rodríguez, María Paz Sanz-Ayán, Juan Ignacio Castillo-Martín, Gustavo Plaza-Manzano

**Affiliations:** 1 Department of Physical Medicine and Rehabilitation, Hospital Universitario 12 de Octubre, 28041 Madrid, Spain; juaniz02@ucm.es (J.I.-G.); paz.sanz@salud.madrid.org (M.P.S.-A.); juanignacio.castillo@salud.madrid.org (J.I.C.-M.); 2 InveCuid, Instituto de Investigación Sanitaria Hospital 12 de Octubre, 28041 Madrid, Spain; 3 Department of Physiotherapy, Universidad Complutense de Madrid, 28040 Madrid, Spain; marconav@ucm.es (M.J.N.-S.); ibailope@ucm.es (I.L.-d.-U.-V.); grabanal@ucm.es (G.R.-R.); gusplaza@ucm.es (G.P.-M.); 4 Grupo InPhysio, Instituto de Investigación Sanitaria del Hospital Clínico San Carlos (IdISSC), 28040 Madrid, Spain; 5 Department of Radiology and Rehabilitation, Universidad Complutense de Madrid, 28040 Madrid, Spain

**Keywords:** longus colli, neck pain, reproducibility, shear-wave elastography, ultrasound imaging

## Abstract

**Highlights:**

**What are the main findings?**

- Standardized SWE delivers reproducible LC stiffness in neck pain.
- Inter- and intra-examiner reliability was good to excellent.

**What are the implications of the main findings?**

- Averaging two measurements improved ICCs and decreased SEM and MDC.
- Further studies can follow this protocol to characterize LC stiffness.

**Abstract:**

Background: Objective, reproducible assessment of deep cervical muscle mechanics is clinically relevant, yet the reliability of shear-wave elastography (SWE) for the longus colli (LC) has not been established. Therefore, the aim of this study was to determine intra- and inter-examiner reliability of LC stiffness measured by SWE under a tightly standardized protocol in patients with mechanical neck pain. Methods: A longitudinal reliability study was conducted. Adults suffering from neck pain for ≥6 months were recruited. Two examiners (with different levels of experience) acquired bilateral LC images using fixed presets. The SWE region of interest covered the full muscle thickness (excluding fascia) to measure the LC shear-wave speed and Young’s modulus. Intraclass correlation coefficients (ICCs), standard error of measurement and minimal detectable changes were computed. Results: Nineteen participants with neck pain completed imaging (left and right sides analyzed). Inter-examiner agreement was good to excellent for single measurements (ICC_3,2_ > 0.818) and improved when averaging two acquisitions (ICC_3,2_ > 0.866). Intra-examiner repeatability was good to excellent for the novel examiner (ICC_3,1_ > 0.891) and excellent for the experienced examiner (ICC_3,1_ > 0.973). No meaningful stiffness differences by sex or side were observed in this sample (*p* > 0.05). Conclusions: A standardized SWE workflow yields reproducible LC stiffness measurements in mechanical neck pain. For longitudinal use, keep a single operator when feasible; in multi-examiner settings, average at least two acquisitions per side to enhance sensitivity to true change.

## 1. Introduction

The Longus Colli muscle (LC) is a deep cervical muscle located on the anterior aspect of the vertebral column, extending from the upper thoracic vertebrae to the atlas, and presents three parts with different origins. The superior oblique portion arises from the anterior tubercles of the transverse processes of the C3 to C5 vertebrae and inserts onto the anterior tubercle of the atlas (C1), the inferior oblique portion originates from the anterior surfaces of the bodies of T1 to T3 vertebrae and attaches to the anterior tubercles of the transverse processes of C5 and C6, and the vertical portion originates from the anterior surfaces of the vertebral bodies of C5 to T3 to insert on the anterior surfaces of the vertebral bodies of C2 to C4 [1,2,3].

This muscle plays a role in cervical movement and motor control. While traditionally considered a stabilizer, its anatomical structure and biomechanical properties suggest a limited capacity for generating significant compression or shear forces. Kennedy et al. [4] indicate that its peak flexion torque is greater in the upper cervical spine but remains small overall, questioning its direct contribution to stability.

From a clinical point of view, dysfunction of the LC is implicated in a broad spectrum of cervical disorders. For instance, in whiplash-associated disorders (WAD), patients commonly show reduced deep-flexor activation with compensatory overactivity of superficial muscles such as the sternocleidomastoid [5], a pattern linked to chronic pain and motor-control deficits [6]. Cervical instability and postural syndromes, which are also closely associated with cervical vertigo, are often characterized by weakness or atrophy of the longus colli, potentially diminishing anterior cervical support [7]. Impaired deep-flexor function can also contribute to cervicogenic headache by disrupting segmental control and increasing upper cervical strain [8,9]. In nonspecific chronic neck pain, altered activation of the deep flexors is frequently observed [10,11], leading to reduced endurance and greater reliance on superficial musculature for head and neck support [12]. Other degenerative conditions, such as cervical spondylosis and disc herniation, may further modify muscle activation through pain inhibition and biomechanical change, potentially reducing longus colli function [12,13,14]. Thus, longus colli tendinitis (a calcific inflammatory entity) typically presents with acute neck pain, restricted motion, and sometimes odynophagia [15]. Finally, after anterior cervical procedures like anterior cervical discectomy and fusion, dissection, inhibition, or scar formation may produce longus colli weakness and post-surgical dysfunction [7,16]. Beyond their diverse clinical labels, these conditions share the altered electromyographic activity and measurable morphological changes in the LC as a common pathophysiological thread. Reduced activation, delayed recruitment, and atrophy or thickness asymmetries have been described across traumatic, degenerative, and post-surgical contexts, suggesting that impaired deep-flexor control is a unifying mechanism rather than a disorder-specific epiphenomenon [11,17,18].

The relevance of stiffness becomes even more apparent in the context of myofascial pain syndrome and myofascial trigger points (MTrPs), which frequently coexist with the conditions outlined above [19,20]. Current diagnostic consensus emphasizes as essential major criteria the identification of a palpable taut band and site-specific stiffness at the MTrP location [21]. By definition, a MTrP is “a hyperirritable palpable nodule within a taut band that can generate local and referred pain, contributing to symptomatology, and that upon stimulation can partially or completely reproduce the patient’s pain pattern” [22]. However, manual palpation, which is by consensus the mainstay for detecting taut bands and localized firmness (as a practical Gold Standard is lacking) [22,23], is inherently subjective, often shows modest agreement with objective stiffness indicators, and is confined to muscles that are anatomically accessible [24,25]. These limitations are especially problematic for deep structures [26], where conventional palpation is not feasible.

Accordingly, there is a clear methodological need to complement neuromuscular assessments with objective, reproducible stiffness measurements in deep cervical musculature [27]. Shear-wave elastography (SWE) is a dynamic ultrasound-based technique that infers tissue stiffness from the propagation of mechanically induced shear waves within the tissue. In SWE, short, focused acoustic “push” pulses generated by the transducer produce a local tissue displacement along the beam axis. This deformation in turn generates transverse shear waves that propagate perpendicular to the initial acoustic beam and spread laterally through the tissue. Ultrafast imaging sequences then track the minute displacements produced by these passing shear waves over time, allowing calculation of the local shear-wave speed (SWS) within the region of interest [28].

From a physical standpoint, SWS is directly related to the shear modulus (G) of the tissue. In an isotropic, nearly incompressible medium with approximately constant density (ρ), the propagation speed of shear waves (V) satisfies V = √(G/ρ), so that G can be estimated as G = ρ·V^2^ [29]. In practical terms, higher SWS and higher derived shear modulus values correspond to greater tissue stiffness, whereas lower values indicate a more compliant, less stiff muscle. Thus, SWE provides quantitative, spatially resolved maps of stiffness that are largely independent of operator-applied compression, can be superimposed on conventional B-mode images for precise ROI placement, and are particularly suitable for obtaining objective, reproducible stiffness measurements in deep muscles [30,31].

Despite the growing use of SWE to characterize cervical muscle mechanical properties, no studies to date have established the reproducibility of SWE-derived stiffness specifically for the LC muscle. This absence of reliability data represents a critical methodological barrier to interpreting existing findings and to designing robust longitudinal or comparative studies involving this deep cervical stabilizer. Developing and testing the reproducibility of imaging-based measures or instrumented approaches that quantify stiffness in the LC would (1) reduce reliance on subjective palpation, (2) enable evaluation of muscles that are not clinically accessible, and (3) provide mechanistic and prognostic biomarkers that can be tracked over time and across interventions. Such metrics could bridge the current evidence gap between observed EMG/morphological abnormalities and the mechanical milieu that likely mediates pain, dysfunction, and recovery in cervical disorders [32]. Therefore, to address this unmet need and provide the first standardized reliability framework for LC stiffness assessment, the aim of this study is to determine the intra- and inter-examiner reliability for calculating the LC muscle stiffness using SWE.

## 2. Materials and Methods

### 2.1. Study Design

Between October 2024 and April 2025, a longitudinal intra- and inter-examiner reliability observational study was conducted within the Radiology service of Hospital Universitario 12 de Octubre, in Madrid. For writing this report, the study adhered to the Reporting Reliability and Agreement Studies (GRRAS) guidelines in order to structure the information provided [33]. A local Ethics Committee provided oversight and approval for the study protocol prior to data collection.

### 2.2. Participants

A cohort of patients referred consecutively from the Traumatology (Orthopedics) Service with neck pain as their primary complaint was prospectively screened for eligibility. Referrals were first pre-screened from the outpatient schedule, and potentially eligible candidates were then contacted and assessed by the research team to confirm the criteria. Inclusion required adults aged 18–65 years with current neck pain of at least 6 months’ duration, a Neck Disability Index (NDI, 0–100) score > 8, and a Numeric Pain Rating Scale (NPRS, 0–10) score > 3 (thresholds commonly used to distinguish symptomatic individuals from asymptomatic comparators [34,35]).

Exclusion criteria comprised any medical, pharmacological, or physiotherapy interventions in the previous 6 months that might influence pain perception or muscle tone; traumatic etiologies (e.g., whiplash-associated disorders, fractures, or fissures); prior cervical surgery; clinical signs of radiculopathy or myelopathy; and other relevant medical conditions, including fibromyalgia or oncologic disease.

Eligibility was verified through clinical interview and record review, and NDI/NPRS were administered in a standardized manner. All candidates received detailed study information and provided written informed consent before any study procedures, and participation did not alter their usual clinical care.

The minimum sample size was estimated following Walter et al.’s guidance using intraclass correlation coefficients (ICCs) [36]. The calculation was set based on a minimum acceptable reliability of 0.7 (as is the minimum threshold for determining good reliability [37]), an expected ICC of 0.9 (since there is no previous reliability data, we used as a references reported ICCs to determine the LC size and shape using B-mode ultrasound [38], 80% statistical power with a two-sided α of 0.05 and an anticipated 10% attrition due to the study’s longitudinal design. Under these assumptions, the minimum number of images to be analyzed was 26. Therefore, the minimum sample size required was 13 participants for intra-examiner reliability (*n* = 26 SWE images in Trial 1 and *n* = 26 in Trial 2) and 26 images for inter-examiner reliability (*n* = 26 images for Examiner 1 and *n* = 26 for Examiner 2).

### 2.3. Examiners

Two examiners with differing expertise acquired and processed all ultrasound data. The senior assessor had more than 10 years of experience in musculoskeletal ultrasound and clinical evaluation, whereas the junior assessor had under 1 year.

Before enrollment began, both completed a structured 2 h calibration on the study device to standardize acquisition and analysis. The session reviewed anatomical landmarks and participant positioning; console configuration with preset locking; probe-handling drills (light transducer pressure, adequate gel, longitudinal B-mode alignment parallel to fiber direction, and orthogonal insonation to limit anisotropy); SWE procedures (activating the mode, placing a region of interest (ROI) that encompassed the entire muscle thickness while excluding fascia, maintaining a stable image for ~10 s prior to freeze), and the capture of both Young’s modulus and shear-wave velocity. Frame acceptance criteria emphasized stability of the elastography/quality map and absence of motion or artifact. Each examiner then practiced the full workflow on a pilot volunteer, obtaining three repeated measures per site and entering values into the study datasheet to rehearse blinding and data entry. Any divergences were discussed immediately, with targeted feedback and a checklist to align ROI placement and artifact rejection decisions before formal data collection.

Data were collected in a dedicated, fully equipped ultrasound room. For each participant, the experienced examiner obtained one image per side, the novice repeated the sequence, the experienced examiner completed a second pass, and the novice performed a final pass (yielding four back-to-back acquisitions per side to limit temporal variability). Between examiners, participants stepped off the table and walked briefly. The starting side for each sequence was randomized.

### 2.4. Shear-Wave Elastography Exam

The US device used for collecting all the images was a Logiq E9 device, using a linear transducer 6–15 MHz ML-6–15-D (General Electric Healthcare, Milwaukee, WI, USA). The console settings were also standard for all the acquisitions.

Participants lay supine in a neutral position with a small towel under the neck to support cervical lordosis; hips and knees were flexed, and the arms rested alongside the body. In addition, all volunteers received instructions to relax their neck muscles to reduce muscle stiffness variability attributable to muscle contraction.

**Figure 1:**
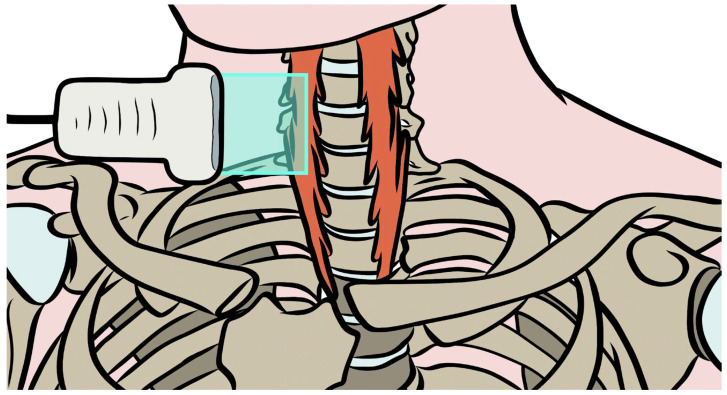
Schematic representation of the ultrasound probe placement along the longitudinal axis of the longus colli muscle. The blue square represents the transducer’s field of view.

The thyroid and cricoid cartilages were palpated, and the transducer was placed in the sagittal plane at the midline between them. Anatomical levels were identified by referencing the cricoid cartilage to C6 and the inferior margin of the thyroid cartilage’s laryngeal prominence to C5 [39]. With the cricoid centered on the screen, the probe was translated laterally across the thyroid gland until the carotid artery was visualized in longitudinal view. At this level, the LC is visualized over the vertebral bodies. Particular care was taken to align the muscle belly strictly perpendicular to the probe’s insonation plane, as any misalignment could introduce artifacts and bias stiffness estimates (Figure 1).

**Figure 2:**
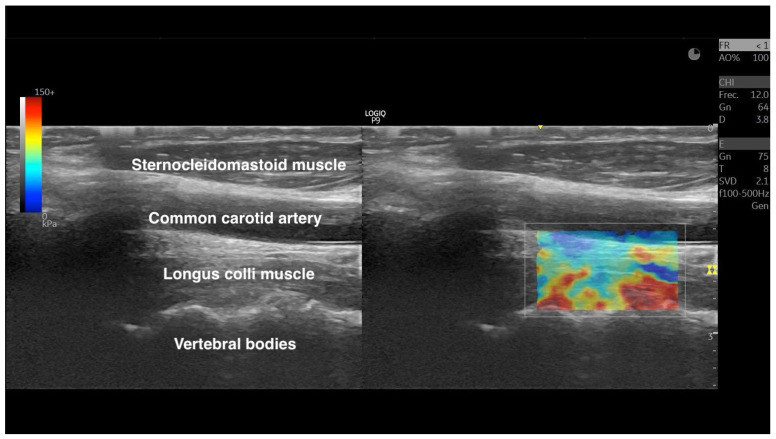
Representative ultrasound of the longus colli. The left panel shows the B-mode image, and the right panel overlays shear-wave elastography, with a color-coded ROI depicting the tissue stiffness distribution. The color map represents the kPa scores; cooler colors indicate lower stiffness and warmer colors indicate higher stiffness, according to the displayed quantitative scale.

To calculate the LC stiffness, the examiners activated the SWE mode and stabilized the image for ~10 s before freezing the image. For measuring, the examiners positioned the region of interest (ROI) to span the full muscle thickness (explicitly excluding surrounding connective and bony tissues) and at least 2 cm of fiber length. After contouring the ROI, the system uses an acoustic radiation force “push” to generate shear waves within the muscle, and the system tracks their propagation using ultrafast imaging. Tissue stiffness is then estimated from the shear-wave propagation speed, which can be reported as shear-wave speed (m/s) and/or converted by the system into an elastic modulus (kPa) under standard assumptions of soft-tissue behavior. Figure 2 shows an illustrative example of SWE image.

### 2.5. Statistical Analysis

Data processing and analysis were performed in SPSS v29 (Armonk, NY, USA) for macOS. All tests were two-tailed, with statistical significance set at *p* < 0.05. The distribution of continuous variables was inspected visually (histograms) and analytically using the Shapiro–Wilk test [40], which evaluates the null hypothesis that the data arise from a normally distributed population. We chose Shapiro–Wilk because of its good power properties in small-to-moderate samples. When *p* > 0.05, the normality assumption was considered acceptable, and sample characteristics were summarized using means and standard deviations; otherwise, medians and interquartile ranges were additionally inspected.

All reliability analyses were conducted following the recommendations for ICCs in reliability studies provided by Koo and Li [37]. The ICC quantifies the proportion of total variance attributable to true between-subject differences, relative to the sum of true variance and measurement error; values closer to 1 therefore indicate that most of the observed variability reflects real differences rather than random error. For test–retest (intra-examiner) reliability, we calculated: (1) the mean of the two trials; (2) the absolute difference between trials as a direct index of dispersion at the individual level; and (3) ICC_3,1_, corresponding to a two-way mixed-effects, single-measurement, consistency model, appropriate when the same fixed examiner is of primary interest and when the focus is on the consistency of scores rather than their absolute agreement. In addition, we estimated the standard error of measurement (SEM) and the minimal detectable change (MDC) to characterize the absolute reliability and the smallest change that can be interpreted as real beyond measurement noise. The SEM was computed as SEM = SD_{mean of trials} × √(1 − ICC), which derives from classical test theory, where measurement error variance is modeled as the complement of reliability. The MDC at the 95% confidence level was then obtained as MDC = SEM × 1.96 × √2, reflecting the propagation of error when comparing two measurements and the z-value for a two-sided 95% confidence interval.

For inter-examiner reliability, we analyzed single measurements and the mean of two measurements separately (as averaging repeated measurements is expected to reduce random error). We computed the grand mean across examiners, the absolute difference between examiners, and ICC_3,2_, a two-way mixed-effects, average-measurement, consistency model that assumes a fixed set of raters and focuses on the reproducibility of mean scores across examiners. SEM and MDC were again derived from the corresponding ICCs.

## 3. Results

From a total of 25 volunteers interested in collaborating with the study, 6 were excluded since they reported being under pharmacological treatment (*n* = 2) or not reaching the minimum NDI or VAS cut-offs (*n* = 4). Therefore, 19 volunteers with neck pain were included in the data collection, analyzing left and right sides of all participants.

Table 1 compares the sociodemographic and clinical characteristics of the sample by gender. Men were older (55.7 ± 6.5 vs. 48.8 ± 8.1 years; Δ = 6.8, 95% CI −1.2 to 14.8; *p* = 0.090) and heavier (72.2 ± 10.8 vs. 63.7 ± 7.2 kg; Δ = 8.5, 95% CI −0.3 to 17.3; *p* = 0.058), but differences did not reach significance; height (*p* = 0.771) and BMI (*p* = 0.199) were also similar. Clinically, women reported significantly longer pain duration (30.0 ± 11.7 vs. 16.3 ± 10.1 months; Δ = 13.6, 95% CI −1.9 to 25.4; *p* = 0.025), while disability (NDI: *p* = 0.229) and pain intensity (NPRS: *p* = 0.137) trended higher in women without statistical significance. Overall, groups were balanced aside from longer symptom chronicity in females.

Table 2 summarizes LC SWS and shear modulus and includes a difference analysis for sex (Group), side (Left/Right), and their interaction. Females showed slightly higher mean stiffness than males (SWS: 6.24 vs. 5.97 m/s; shear modulus: 115.8 vs. 108.3 kPa), and the right side was marginally higher than the left in both sexes, but none of these differences were statistically significant. For SWS, Group: F = 0.986, *p* = 0.328, ηp^2^ = 0.028; Side: F = 0.637, *p* = 0.430, ηp^2^ = 0.018; Group × Side: F = 0.176, *p* = 0.677, ηp^2^ = 0.005. For shear modulus, Group: F = 0.624, *p* = 0.435, ηp^2^ = 0.018; Side: F = 0.743, *p* = 0.395, ηp^2^ = 0.021; Group × Side: F = 0.051, *p* = 0.823, ηp^2^ = 0.001. Effect sizes were trivial to small, indicating bilateral symmetry and no sex-specific differences in this sample; pooling by side and sex appears defensible for primary analyses.

Inter-examiner reliability (reported for single measurements and for the mean of two measurements) is detailed in Table 3. Agreement between examiners was good to excellent. For single measurements, ICC_3,2_ was 0.818 for SWS and 0.849 for shear modulus; averaging two measurements increased ICCs to 0.866 and 0.883, respectively. Between-examiner bias was negligible (all mean differences non-significant; *p* ≥ 0.280). Precision improved with averaging: absolute error decreased (SWS 0.39→0.32 m/s; shear modulus 12.5→10.8 kPa), SEM fell (0.37→0.30 m/s; 11.5→9.5 kPa), and MDC narrowed (1.03→0.83 m/s; 31.8→26.4 kPa). Expressed relative to the grand means, SEM was ~5–6% for speed and ~8–10% for shear modulus, with MDCs ~13–17% (SWS) and ~23–29% (shear modulus). Overall, results indicate small measurement error, no systematic examiner effect, and a modest gain in reliability and detectability of change when averaging two acquisitions.

Finally, intra-examiner reliability data are summarized in Table 4. Test–retest agreement was excellent for both examiners, with consistently negligible trial-to-trial bias (all mean differences non-significant: *p* = 0.928–0.987 for SWS; *p* = 0.919–0.951 for shear modulus). ICC_3,1_ values were high throughout, but higher for the experienced examiner—especially for shear modulus (novice: 0.906–0.891 for SWS; 0.974–0.973 for shear modulus; experienced: 0.974–0.973 for shear modulus and 0.906–0.891 for SWS mirrored, per columns). Precision metrics favored the experienced examiner: absolute error and SEM were smaller (SWS SEM 0.14 vs. 0.26 m/s; shear modulus SEM 4.8 vs. 9.3 kPa), yielding tighter MDCs (SWS 0.38 vs. 0.71 m/s; shear modulus 13.4 vs. 25.7 kPa). Expressed relative to the means, SEMs were ~2–4% (experienced) vs. ~4–8% (novice), with MDCs ~6–12% vs. ~11–22%. Overall, single-operator repeatability was strongest for shear modulus and improved with greater examiner experience, and there was no evidence of systematic drift between trials.

## 4. Discussion

SWE is an ultrasound technique that uses focused acoustic radiation force to generate transverse shear waves in tissue; by tracking their speed, the scanner derives quantitative maps of stiffness (shear modulus) that are automatically coregistered with B-mode imaging and displayed as color elastograms in m/s or kPa [41]. Compared with compression elastography, SWE is more objective and reproducible, enabling direct, operator-independent assessment of mechanical properties [30]. In the musculoskeletal system, shear waves travel faster in stiffer or contracted tissue and along tendon/muscle fiber axes, allowing SWE to complement grayscale and Doppler ultrasound for early detection, grading, and follow-up of pathology across tendons, muscles, peripheral nerves, ligaments, and selected soft-tissue masses. Emerging evidence highlights its value for monitoring healing, staging chronic disease, and potentially predicting tendon failure risk, while ongoing advances (e.g., continuous-wave and 3D SWE) broaden clinical utility [28,30,31,41].

However, SWE also has practical limitations. Penetration is limited in deep structures and may require a gel standoff; measurements are sensitive to probe pressure, insonation angle, and tissue anisotropy, so small misalignments can alter wave speed; some systems restrict ROI size/shape and impose pauses between acquisitions, complicating truly dynamic studies; image quality can degrade in the presence of fluid, producing voids in the elastogram; and results depend on device presets and vendor-specific algorithms, risking spurious values if scales are misconfigured and reducing cross-platform comparability [28,30,31,41].

Since these factors affect reproducibility and clinical interpretation, fully exploiting SWE for LC assessment first requires quantifying its measurement reliability, as the modality is operator-dependent. It is also necessary to determine the minimal changes or differences that distinguish true alterations in muscle stiffness (whether observed over time due to interventions or natural progression or between groups) from measurement error. Establishing these parameters is essential both for accurately interpreting previously published studies, many of which lack such analyses, and for designing future research, including cross-sectional case–control comparisons and longitudinal evaluations of therapeutic interventions on LC stiffness. Accordingly, this study aims to provide a detailed, reproducible SWE protocol for calculating LC stiffness and to report intra- and inter-examiner reliability data to support future cross-sectional and longitudinal studies.

Focusing on reliability, measurements showed consistently good-to-excellent agreement between examiners with negligible systematic bias; averaging two acquisitions further reduced random error and improved the ability to detect true change. Within-examiner repeatability was excellent for both shear-wave speed and shear modulus, with tighter stability under the more experienced operator. Error indices were small relative to the signal, indicating robust precision under the standardized protocol. Practically, longitudinal monitoring should prioritize a single operator when feasible, while multi-examiner settings benefit from averaging at least two acquisitions per side to optimize reproducibility and sensitivity to change.

While a direct reliability estimates comparison with previous studies is not possible, as this is the first study evaluating the SWE procedure, an indirect benchmark can be drawn from B-mode ultrasonography of LC morphology. For instance, in asymptomatic populations, Jeong et al. showed excellent intra- and inter-rater reliability for B-mode measurements of LC thickness and cross-sectional area when probe pressure was standardized (ICC ≈ 0.96–0.99), underscoring the value of tight acquisition control [42], and Nagai et al. [43] reported good-to-excellent intra-rater reliability for B-mode thickness/CSA outcomes (ICC 0.715–0.890) but only moderate repeatability for SCM shear-wave elastography stiffness (ICC = 0.554), highlighting that SWE can be less stable than morphology in healthy samples. However, the LC was not tested using SWE.

By contrast, in patient populations, Javanshir et al. [38] found LC morphology to be consistently reproducible in chronic mechanical neck pain: within-day intra-examiner ICCs were ≥0.86 and between-day ICCs ≥ 0.81 for CSA, anterior–posterior and lateral dimensions, and shape ratio. Mohseni et al. [44] demonstrated good-to-excellent reliability in unilateral cervical disc herniation for LC, multifidus, and semispinalis cervicis dimensions (within-day intra-/inter-rater ICC 0.82–0.96; between-day intra-rater ICC 0.75–0.89), with acceptable SEM/MDC values suitable for longitudinal monitoring.

### Limitations

A key limitation is that our sample included only individuals with mechanical neck pain. Because neck pain spans multiple subtypes (i.e., pain associated with mobility deficits, headaches, radiating pain, and movement coordination impairments [45]), the muscle’s stiffness characteristics may differ depending on the underlying etiology. Another limitation is that measurements were taken at a single, generalized site using one ultrasound system. Future research should target specific locations (e.g., myofascial trigger points) and include multiple ultrasound brands/models to strengthen external validity. Finally, although stiffness values were reported and compared by sex and side, the study was powered for reliability rather than group contrasts. As such, it may lack sufficient statistical power to detect small-to-moderate mean differences or associations; these findings should be interpreted cautiously and confirmed in larger, hypothesis-driven samples.

## 5. Conclusions

Under a tightly standardized SWE protocol for assessing the LC stiffness, the obtained results demonstrated good-to-excellent inter-examiner agreement with negligible bias and excellent within-examiner repeatability for both shear-wave speed and shear modulus, with precision improving further when two acquisitions were averaged. Error indices (SEM/MDC) were small relative to the signal, supporting the use of these measures for monitoring true change over time. Practically, longitudinal follow-up should prioritize a single operator when feasible; in multi-examiner contexts, averaging at least two measurements per side enhances reproducibility and sensitivity. No meaningful stiffness differences by sex or side were detected, suggesting pooling is defensible in similar samples. Collectively, these findings establish a reproducible framework for LC stiffness assessment and provide reliability parameters to inform sample-size planning and interpretation in future cross-sectional and interventional studies.

## Figures and Tables

**Table 1:** Descriptive analyses of demographic and clinical characteristics of the sample.

Variables	Males (*n* = 6)	Females (*n* = 13)	Gender Difference (95% CI)
Demographics			
Age, years	55.7 ± 6.5	48.8 ± 8.1	6.8 (−1.2; 14.8) *p* = 0.090
Weight, kg	72.2 ± 10.8	63.7 ± 7.2	8.5 (−0.3; 17.3) *p* = 0.058
Height, m	1.65 ± 0.07	1.64 ± 0.06	0.01 (−0.05; 0.07) *p* = 0.771
BMI, kg/m^2^	26.4 ± 3.6	23.8 ± 4.1	2.6 (−1.51; 6.72) *p* = 0.199
Clinical Characteristics			
Pain duration, months	16.3 ± 10.1	30.0 ± 11.7	13.6 (−1.9; 25.4) *p* = 0.025
Neck Disability Index, 0–100	29.3 ± 12.8	36.4 ± 10.8	7.1 (−4.9; 19.0) *p* = 0.229
Numeric Pain Rating Scale, 0–10	6.2 ± 1.3	7.0 ± 0.91	0.8 (−0.3; 1.9) *p* = 0.137

Abbreviations: BMI, Body Mass Index.

**Table 2:** Longus colli stiffness: Descriptive analysis and differences between genders and sides.

Gender	Side	Shear-Wave Speed (m/s)	Shear Modulus (kPa)
Descriptive shear-wave elastography scores *			
Males	Mean	5.97 ± 0.93	108.3 ± 30.3
	Left (*n* = 6)	5.83 ± 1.04	102.3 ± 34.2
	Right (*n* = 6)	6.10 ± 0.88	114.4 ± 27.7
Females	Mean	6.24 ± 0.68	115.8 ± 24.7
	Left (*n* = 13)	6.16 ± 0.79	113.7 ± 28.4
	Right (*n* = 13)	6.31 ± 0.57	117 9 ± 21.2
Differences			
Gender	F	0.986	0.624
	*p* Value	0.328	0.435
	$\eta_{p}^{2}$	0.028	0.018
Side	F	0.637	0.743
	*p* Value	0.430	0.395
	$\eta_{p}^{2}$	0.018	0.021
Gender × Side	F	0.176	0.051
	*p* Value	0.677	0.823
	$\eta_{p}^{2}$	0.005	0.001

* Reported values are calculated as the mean average of both trials from both examiners.

**Table 3:** Inter-examiner reliability analyses to determine general longus colli muscle stiffness.

Reliability Estimates	Shear-Wave Speed (m/s)		Shear Modulus (kPa)	
	Novice Examiner (*n* = 38 Images)	Experienced Examiner (*n* = 38 Images)	Novice Examiner (*n* = 38 Images)	Experienced Examiner (*n* = 38 Images)
Single Measurements				
Mean	6.24 ± 0.88	6.06 ± 0.86	117.2 ± 29.0	109.7 ± 30.0
Difference	0.15 (−0.24; 0.55) *p* = 0.442		6.4 (−7.1; 19.8) *p* = 0.349	
Absolute Error	0.39 ± 0.58		12.5 ± 18.7	
ICC_3,2_, 0–1	0.818 (0.649; 0.905)		0.849 (0.710; 0.922)	
SEM	0.37		11.5	
MDC	1.03		31.8	
Mean Average of 2 Measurements				
Mean	6.24 ± 0.80	6.07 ± 0.83	116.9 ± 26.6	109.9 ± 29.1
Difference	0.17 (−0.20; 0.54) *p* = 0.362		7.0 (−5.8; 19.7) *p* = 0.280	
Absolute Error	0.32 ± 0.49		10.8 ± 16.0	
ICC_3,2_, 0–1	0.866 (0.742; 0.930)		0.883 (0.775; 0.939)	
SEM	0.30		9.5	
MDC	0.83		26.4	

ICC: Intraclass correlation coefficients; MDC: Minimal Detectable Changes; SEM: Standard Error of Measurement.

**Table 4:** Test–retest reliability analyses to determine general longus colli muscle stiffness.

Reliability Estimates	Novice Examiner				Experienced Examiner			
	Shear-Wave Speed (m/s)		Shear Modulus (kPa)		Shear-Wave Speed (m/s)		Shear Modulus (kPa)	
	Trial 1	Trial 2	Trial 1	Trial 2	Trial 1	Trial 2	Trial 1	Trial 2
Mean	6.24 ± 0.88	6.23 ± 0.78	117.2 ± 29.0	116.6 ± 27.1	6.06 ± 0.86	6.07 ± 0.82	109.7 ± 30.0	110.2 ± 29.0
Difference	0.00 (−0.4; 0.4) *p* = 0.987		0.7 (−12.1; 13.5) *p* = 0.919		0.01 (−0.36; 0.40) *p* = 0.928		0.4 (−13.0; 13.9) *p* = 0.951	
Absolute Error	0.28 ± 0.40		10.0 ± 14.4		0.20 ± 0.16		7.5 ± 5.8	
ICC_3,1_, 0–1	0.906 (0.819; 0.951)		0.891 (0.790; 0.943)		0.974 (0.950; 0.987)		0.973 (0.949; 0.986)	
SEM	0.26		9.3		0.14		4.8	
MDC	0.71		25.7		0.38		13.4	

ICC: Intraclass correlation coefficients; MDC: Minimal Detectable Changes; SEM: Standard Error of Measurement. Each trial corresponds to the acquisition and measurement of *n* = 38 images.

## Data Availability

The datasets used and/or analyzed during the current study are available from the corresponding author on reasonable request.
